# Longitudinal brain atrophy and mortality among people living in homelessness and precarious housing: A brief report of a longitudinal study

**DOI:** 10.1371/journal.pone.0340056

**Published:** 2026-02-18

**Authors:** Jacob L. Stubbs, Andrea A. Jones, Kristina M. Gicas, Thalia S. Field, Manraj K. S. Heran, A. Talia Vertinsky, Donna J. Lang, Wayne Su, Allen E. Thornton, G. William MacEwan, Olga Leonova, Alexander Rauscher, Alasdair M. Barr, William G. Honer, William J. Panenka

**Affiliations:** 1 Department of Psychiatry, University of British Columbia, Vancouver, BC, Canada; 2 British Columbia Mental Health and Substance Use Services Research Institute, Vancouver, BC, Canada; 3 Division of Neurology, University of British Columbia, Vancouver, BC, Canada; 4 Department of Psychology, University of the Fraser Valley, Abbotsford, BC, Canada; 5 Department of Radiology, University of British Columbia, Vancouver, BC, Canada; 6 Department of Psychology, Simon Fraser University, Burnaby, BC, Canada; 7 Department of Pediatrics, University of British Columbia, Vancouver, BC, Canada; 8 Department of Anesthesiology, Pharmacology, and Therapeutics, University of British Columbia, Vancouver, BC, Canada; 9 British Columbia Neuropsychiatry Program, Vancouver, BC, Canada; Cardiff University, UNITED KINGDOM OF GREAT BRITAIN AND NORTHERN IRELAND

## Abstract

**Background:**

People living in homelessness or precarious housing experience more health challenges and earlier mortality than the general population. We characterized longitudinal changes in magnetic resonance imaging (MRI) measures of brain atrophy among people living without stable housing, evaluated risk factors associated with longitudinal atrophy, and assessed whether atrophy was associated with mortality.

**Methods:**

Data was collected as part of an ongoing longitudinal observational study of people living without stable housing in Vancouver, Canada. We included 307 participants with two-or-more brain MRI scans over an average of 7.0 years. We evaluated select risk factors for brain atrophy and assessed whether brain atrophy was associated with mortality during the study.

**Results:**

Across 307 participants and 1,173 brain MRI scans, alcohol dependence (*β* = −0.053, 95% confidence interval [CI] −0.075 to −0.032, *p* < 0.0001) and higher cardiovascular risk scores (*β* = −0.021, 95% CI −0.029 to −0.012, *p* < 0.0001) were associated with more rapid brain atrophy over time, and history of moderate or severe traumatic brain injury was associated with more atrophy at baseline (*β* = −0.27, 95% CI −0.46 to −0.075, *p* = 0.0075). More brain atrophy at baseline was associated with mortality during the study, adjusting for age, sex, and other comorbidities (Hazard Ratio [HR]_Quartile1_ = reference; HR_Q2_ = 2.37, *p* = 0.035; HR_Q3_ = 4.01, *p* = 0.00032; HR_Q4_ = 4.91, *p* < 0.0001).

**Conclusions and relevance:**

Alcohol dependence, cardiovascular risk, and traumatic brain injury may represent particularly important risk factors for brain atrophy, and brain atrophy is associated with mortality among people living without stable housing. Targeting these modifiable risk factors may improve brain health and functioning outcomes among people living without stable housing.

## Introduction

People living in homelessness and precarious housing have poorer health and higher mortality compared to the general population [1–3]. There is a disproportionately high prevalence of risk factors for neurological health, including substance dependence, infectious disease, and traumatic brain injury among people living without stable housing [4,5]. These factors likely contribute to early onset of age-related pathologies, including cognitive dysfunction and geriatric syndromes [6–8]. Understanding relationships between neurologic risk factors, brain structure, and health outcomes is an important part of pursuing health equity and addressing the health disparities experienced by people living without stable housing. Despite many risk factors for brain impairment, there are no studies that have used longitudinal quantitative neuroimaging to evaluate brain structure and health among these individuals. In this study, we evaluated risk factors associated with longitudinal trajectories of brain atrophy and assessed whether the degree of brain atrophy was associated with mortality among people living in homelessness and precarious housing.

## Methods

### Participants and neuroimaging

Participants were recruited from single-room occupancy hotels, a community court, and a local hospital as part of a prospective longitudinal study of people living in homelessness and precarious housing in an impoverished neighbourhood of Vancouver, Canada [9]. Criteria for enrolment included being age 18 or older and the ability to speak English. No specific exclusion criteria were applied. Participant demographics and health challenges were similar to other studies on homelessness [7]. Recruitment for the study occurred between Nov. 13^th^, 2008 and Feb. 6^th^, 2017.

Participants were included in this analysis if they had two or more T1-weighted brain magnetic resonance imaging (MRI) scans that were of sufficient quality to be analyzed. Whole-brain atrophy was estimated using tissue-to-intracranial volume ratio. All scans were acquired on the same scanner, with full acquisition and processing details described in the Supplementary Methods. For reference, we also included data from an open access sample of participants recruited from the general population [10], and we used identical processing and analysis steps as in the precarious housing sample.

### Risk factor and mortality data

We assessed the association between select risk factors and longitudinal patterns of brain atrophy. Risk factors included substance dependence diagnoses, history of intravenous drug use, human immunodeficiency virus (HIV) status, Framingham risk score (a measure of 10-year atherosclerotic cardiovascular disease risk), and history of traumatic brain injury. Baseline comorbidities were quantified with the Charlson comorbidity index. Details of each assessment are described in the Supplementary Methods. We then assessed whether the extent of brain atrophy at baseline was associated with mortality during the study period.

### Statistical analysis

We used linear mixed-effects models to characterize longitudinal changes in brain atrophy over time and across regions of interest. All region of interest analyses were corrected for multiple comparisons to a false discovery rate of *p* < 0.05. Next, we used linear mixed-effects models to evaluate what baseline risk factors were associated with rates of longitudinal brain atrophy. Finally, we used Kaplan-Meier curves and Cox proportional-hazards regression to evaluate whether more brain atrophy on baseline MRI scan was associated with mortality during the study period (where the first quartile (>75%) represents the least amount of brain atrophy and the fourth quartile (<25%) represents the most). Details of all analyses are described in the Supplementary Methods. All statistical analyses were done using R version 4.0.3 [11].

### Ethical approval and data sharing

The Hotel Study was approved by the Research Ethics Board at the University of British Columbia (H08-00521) and the general population comparison sample was approved by the Cambridgeshire 2 Research Ethics Committee (10/H0308/50). Written informed consent was acquired for all precariously housed participants starting November 13^th^, 2008. Data from the precariously housed sample cannot be made publicly available due to possible privacy breaches and other ethical and legal obligations to the study participants. Requests for collaboration or data access may be made to the corresponding author or the Research Ethics Board of the University of British Columbia. Data from the general population reference sample is publicly available [10].

## Results

We included 307 homeless or precariously housed participants with a total 1,173 brain MRI scans (median number of scans per participant = 3, range = 2–8). The average age of the sample was 42.1 (standard deviation [SD]=11.0) and 77.5% were male. The mean follow-up time was 7.0 years (SD = 4.4, max. = 14.3). Baseline characteristics of the study sample are outlined in the S1 Table in S1 File.

**Fig 1:**
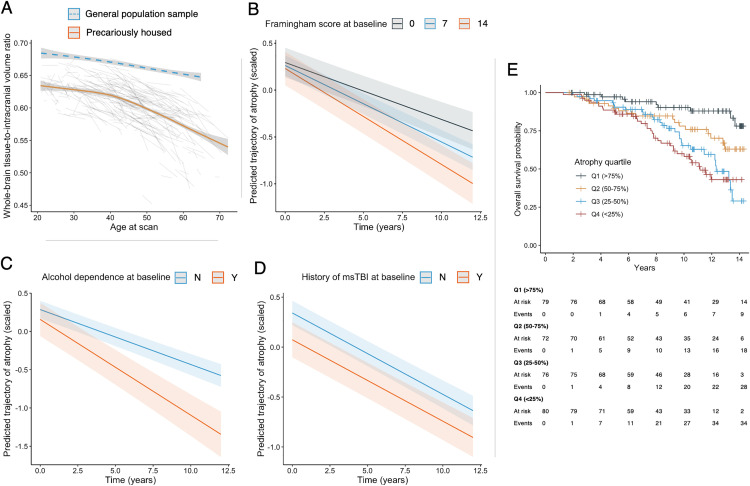
Trajectories of MRI measures of brain structure among individuals living in precarious housing and association with mortality. **(A)** Whole-brain tissue-to-intracranial volume ratio as a function of age for the precariously housed sample (orange line = average trajectory of the precariously housed sample + 95% confidence interval; light grey lines = individual trajectories of precariously housed participants; blue dashed line = reference estimate derived from general population sample + 95% confidence interval). **(B)** Predicted atrophy trajectory over time in the precariously housed sample stratified by baseline Framingham Cardiovascular Risk score. **(C)**. Predicted atrophy trajectory over time in the precariously housed sample stratified by alcohol dependence at baseline. **(D)** Predicted atrophy trajectory over time in the precariously housed sample study stratified by history of moderate-severe TBI (msTBI). **(E)** Kaplan-Meier curve stratified based on baseline brain atrophy. Q1 (>75%) represents those with the least brain atrophy at baseline and Q4 (<25%) represents those with the most atrophy.

There was significant brain atrophy over time among participants living in homelessness or precarious housing (*β* = −0.072, 95% confidence interval [CI] −0.081 to −0.063, *p* < 0.0001). The rate of atrophy increased with older age, especially after middle age (Fig 1A), and rates of atrophy differed across cortical and subcortical regions (S1–S3 Figs in S1 File). Previous work has shown that this rate is disproportionately higher than what would be expected in the general population [12], and a general population reference line is shown in Fig 1A.

Among the risk factors we assessed, alcohol dependence (*β* = −0.053, 95% CI −0.075 to −0.032, *p* < 0.0001) and higher cardiovascular risk scores (*β* = −0.021, 95% CI −0.029 to −0.012, *p* < 0.0001) at baseline were associated with more rapid atrophy during the study, adjusting for age, sex, and baseline comorbidities (Fig 1B and 1C; S2 and S3 Tables in S1 File). History of moderate or severe traumatic brain injury was associated with more atrophy at baseline, adjusting for age, sex, and baseline comorbidities (Fig 1D; *β* = −0.27, 95% CI −0.46 to −0.075, *p* = 0.0075). Patterns of atrophy differed between risk factors across cortical and subcortical regions (S4 and S5 Figs in S1 File).

Eighty-nine participants (29.0%) had died during the study at the time of analysis. Baseline atrophy across quartile is shown in the S6 Fig in S1 File. Survival during the study stratified by quartile of baseline brain atrophy is shown in Fig 1E. More brain atrophy at baseline was associated with a higher risk of mortality during the study (Hazard Ratio [HR]_Quartile1_ = reference; HR_Q2_ = 2.37, *p* = 0.035; HR_Q3_ = 4.01, *p* = 0.00032; HR_Q4_ = 4.91, *p* < 0.0001), and results were similar when adjusting for age, sex, and baseline comorbidities (S3 Table in S1 File).

## Discussion

Our work provides quantitative evidence that supports a neurobiological basis for the early decline in health and functioning experienced by people living without stable housing. This study builds on our previous work, and the work of others, to show that people living without stable housing experience earlier and more rapid brain atrophy, faster cognitive decline, more age-related syndromes, and greater mortality than would be expected in the general population [1,6–8,12]. Death among people living in homelessness are generally due to physical illness, injury, and overdose [1,13]. Our work highlights the strong association between brain atrophy and health decline among these individuals. The strongest risk factors for brain atrophy that we found (alcohol dependence, cardiovascular risk, and traumatic brain injury) are similar to those seen in dementia studies in the general population [14]. Notably, however, individuals living without stable housing experience these brain and functional changes at considerably younger ages than is seen in the general population.

This is the first study, to our knowledge, that has used quantitative neuroimaging to evaluate longitudinal brain atrophy among people living without stable housing. However, this study has limitations. First, there are risk factors that we were unable to evaluate. For example, we did not have sufficient data to evaluate the role of drug overdoses which are a risk factor for hypoxic brain injury and death. Assault is also common among people living in homelessness and the relationship between assault and brain health should be evaluated in future studies. Second, there are complementary brain imaging measures that we did not assess but which could lend further insight into the brain changes experienced by people living without stable housing. There are also other measures of brain function and pathology, such as EEG and CSF markers, that could be useful areas of future work to expand on our findings. Lastly, while we covaried for other medical comorbidities in our mortality analysis, we did not include other specific risk factors for mortality (such as psychosis) that can contribute to mortality risk. We also did not evaluate specific causes of death for participants in the present study. Future work could evaluate associations between brain atrophy and specific causes of death to further expand on our findings.

In summary, people living in homelessness and precarious housing experience significant brain atrophy over time and more brain atrophy is associated with mortality. While further work is needed to fully characterize the brain health challenges of people living without stable housing, our study highlights the need for screening and early interventions that support the brain health of these individuals. Prevention and treatment strategies relating to alcohol use, cardiovascular risk, and traumatic brain injury may be helpful for improving the long-term brain health of people living without stable housing. Support services available to older adults in the general population may be beneficial to people living without stable housing if available at a younger age. This is underscored by data that suggests the median age of death among people living in homelessness is less than 65 years old [15].

## Supporting information

S1 File Supporting files contains eTable 1–3 and eFigure 1–5. (DOCX)
