# Supplementary material for: Longitudinal brain atrophy and mortality among people living in homelessness and precarious housing: A brief report of a longitudinal study
Source: PLoS One. 2026 Feb 18;21(2):e0340056. doi: 10.1371/journal.pone.0340056 (PMC12915904; doi:10.1371/journal.pone.0340056)
Supplement: S1 File — (DOCX) [file pone.0340056.s001.docx]

**Supplementary Materials**

Page 2. Supplementary Methods

Page 3. eTable 1. Demographic and clinical characteristics of the study sample.

Page 4. eFigure 1. Longitudinal change across regions of interest.

Page 5. eFigure 2. Longitudinal rates of change across subcortical regions of interest.

Page 5. eFigure 3. Longitudinal rates of change across cortical regions of interest.

Page 6. eTable 2. Associations between risk factors and longitudinal atrophy.

Page 6. eFigure 4. Rates of atrophy in those with alcohol dependence.

Page 7. eFigure 5. Rates of atrophy in those with higher Framingham risk scores.

Page 7. eTable 3. Association between baseline atrophy and mortality during the study period.

Page 8. Supplementary references

**Supplementary Methods**

*Neuroimaging acquisition and processing*

Hotel Study scans were acquired using the 3T Philips Achieva scanner using an eight-channel SENSE head coil. High resolution 3D T1-weighted FFE sagittal images were acquired with TE = 3.7 ms, TR = 8.1 ms, flip angle 8°, FOV = 256mm × 256mm, acquisition matrix = 256 × 250, reconstruction matrix = 256 × 256, voxel spacing = 1.0 mm × 1.0 mm × 1.0 mm, 190 contiguous slices, slice thickness = 1 mm, gap = 0, SENSE = 1, scan duration = 7:23 min.

T1-weighted scans were processed with FreeSurfer version 6.0 according to the FreeSurfer longitudinal pipeline.^1^ Cortical thickness was extracted according to the Desikan-Killiany atlas^2^ and subcortical volumes were exported according to the standard FreeSurfer subcortical segmentation atlas.^3^ Tissue-to-intracranial volume ratio was defined as supratentorial tissue volume divided by estimated intracranial volume. Region of interest figures were produced using the R package “*ggseg*”.^4^

*Measures*

Substance dependence diagnoses were assigned by a team of study psychiatrists using the Best Estimate Clinical Evaluation and Diagnosis process,^5^ history of intravenous drug use was assessed by self-report at study entry, and HIV infection was assessed using baseline serology. Framingham risk score was calculated using the 10-year cardiovascular disease risk model made available by the Framingham Heart Study. History of traumatic brain injury (TBI) was assessed with a composite measure compiled from three sources to minimize the number of participants with missing data. Participants were classified as having any history of TBI if they endorsed receiving a blow to the head and a period of self-reported loss of consciousness on a baseline medical history interview or the Brain Injury Screening Questionnaire (BISQ),^6^ of if they had evidence of TBI visible on structural MRI. Participants were classified as having a history of moderate or severe TBI if they endorsed a blow to the head and loss of consciousness for more than 30 minutes, or if they had evidence of TBI visible on structural MRI. Ascertainment of trauma-related pathology on MRI was done through consensus diagnosis by fellowship trained neuroradioloists (TV and MHR), a stroke neurologist (TSF), and a neurologist specializing in TBI (WP).

*Statistical analysis*

We described longitudinal trajectories of brain atrophy as a function of age using general additive models. To evaluate what baseline factors were associated with the rate of atrophy over time, we used linear mixed-effects models with fixed effects of time, predictor of interest, and a predictor × time interaction term to screen for variables to include in adjusted models. Participant was included as a random effect. We then took variables or their interaction terms that had a *p*-value of < 0.10 in univariable models and added them into adjusted models, with baseline age, sex, and baseline comorbidities forced into each intermediate and final model. We used multiple imputation to impute missing risk factor data, running our models using both the observed data with missingness and the imputed datasets. As the model results were not substantially different in the nature or direction of the findings, we report our results using the imputed dataset to retain the full sample size. For variables that were significantly associated with more rapid decline in whole-brain atrophy, we evaluated differential effects in the rate of change across regions of interest.

To evaluate whether brain atrophy at baseline was associated with mortality during the study period we used Kaplan-Meier curves and Cox proportional-hazards regression. First, we divided participants into quartiles based on their baseline tissue-to-intracranial volume ratio (where Q1 (>75%) represents the least amount of brain atrophy and Q4 (<25%) represents the most). We used these quartiles as the strata for a Kaplan-Meier curve. We then included these strata in Cox proportional-hazards regression to evaluate whether more brain atrophy was statistically significantly associated with mortality compared to the least (Q1) amount of brain atrophy. We then repeated our analyses while covarying for age at baseline, sex, and baseline comorbidities. All model assumptions were assessed and met.

**Supplementary Results**

**eTable 1. Demographic and clinical characteristics of the study sample.**

| **Characteristic** | **n** | **N** | **%** |
| --- | --- | --- | --- |
| Substance dependence diagnoses |  |  |  |
| Alcohol, n/N (%) | 57 | 307 | 18.6 |
| Cannabis, n/N (%) | 112 | 307 | 36.5 |
| Cocaine, n/N (%) | 182 | 307 | 59.3 |
| Methamphetamine, n/N (%) | 100 | 307 | 32.6 |
| Opiate, n/N (%) | 162 | 307 | 52.8 |
| History of intravenous drug use, n/N (%) | 244 | 305 | 80.0 |
| HIV infection, n/N (%) | 43 | 289 | 14.9 |
| Traumatic brain injury history |  |  |  |
| History of any TBI with LOC | 152 | 257 | 59.1 |
| History of mild TBI with LOC | 121 | 257 | 47.1 |
| History of moderate or severe TBI (LOC > 30 min.), or evidence of TBI visible on MRI | 83 | 266 | 31.2 |
| TBI = traumatic brain injury; LOC = loss of consciousness; MRI = magnetic resonance imaging | | | |


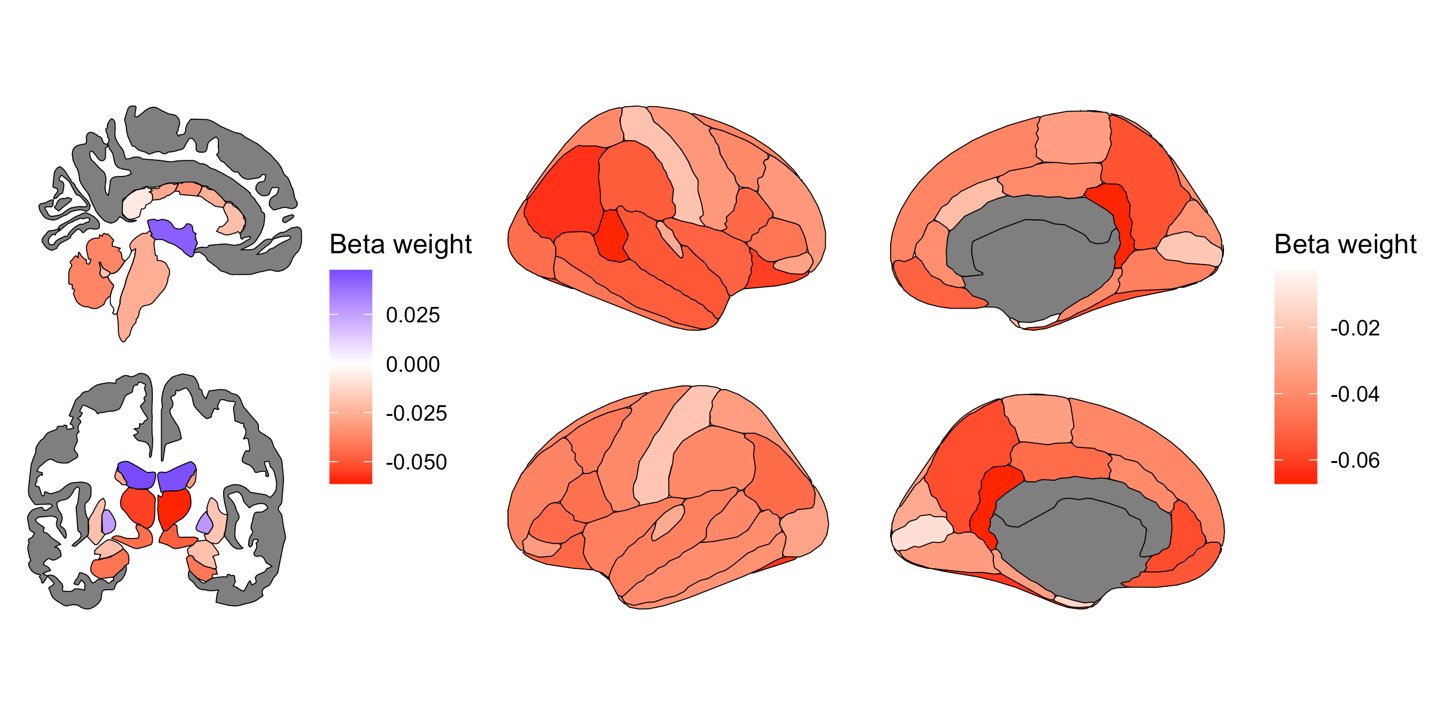


**eFigure 1. Longitudinal change across regions of interest.** Predicted longitudinal rates of change across subcortical (left) and cortical (right) regions of interest. Beta weights denote the slope over time of the regional volume as derived using a linear mixed-effect regression model. Cool colours represent volume increase and hot colours represent volume decrease. Exact values are reported in Supplementary Figures 2 and 3.


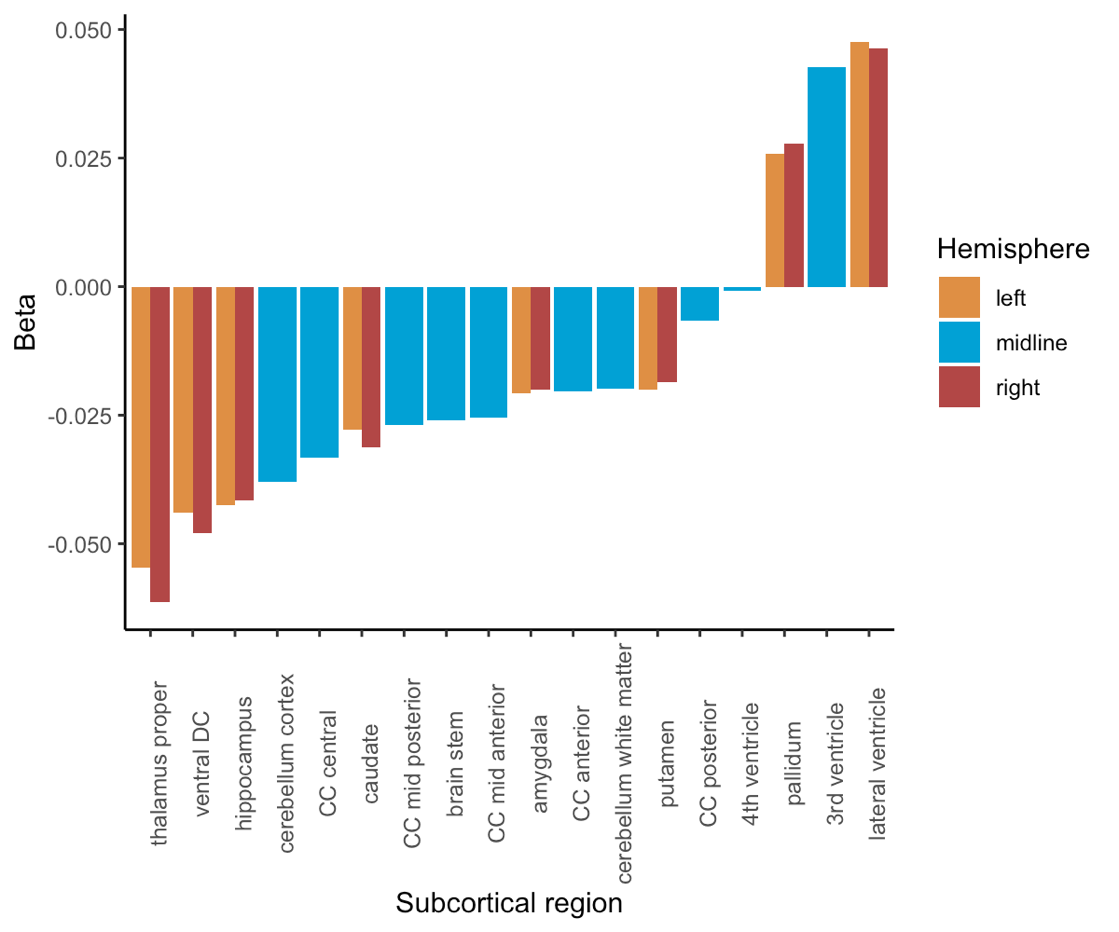


**eFigure 2. Longitudinal rates of change across subcortical regions of interest.** All were statistically significant at *p*_FDR_ < 0.05 except for the 4^th^ ventricle (*p*_FDR_ = 0.89) and the posterior portion of the corpus callosum (*p*_FDR_ = 0.099).


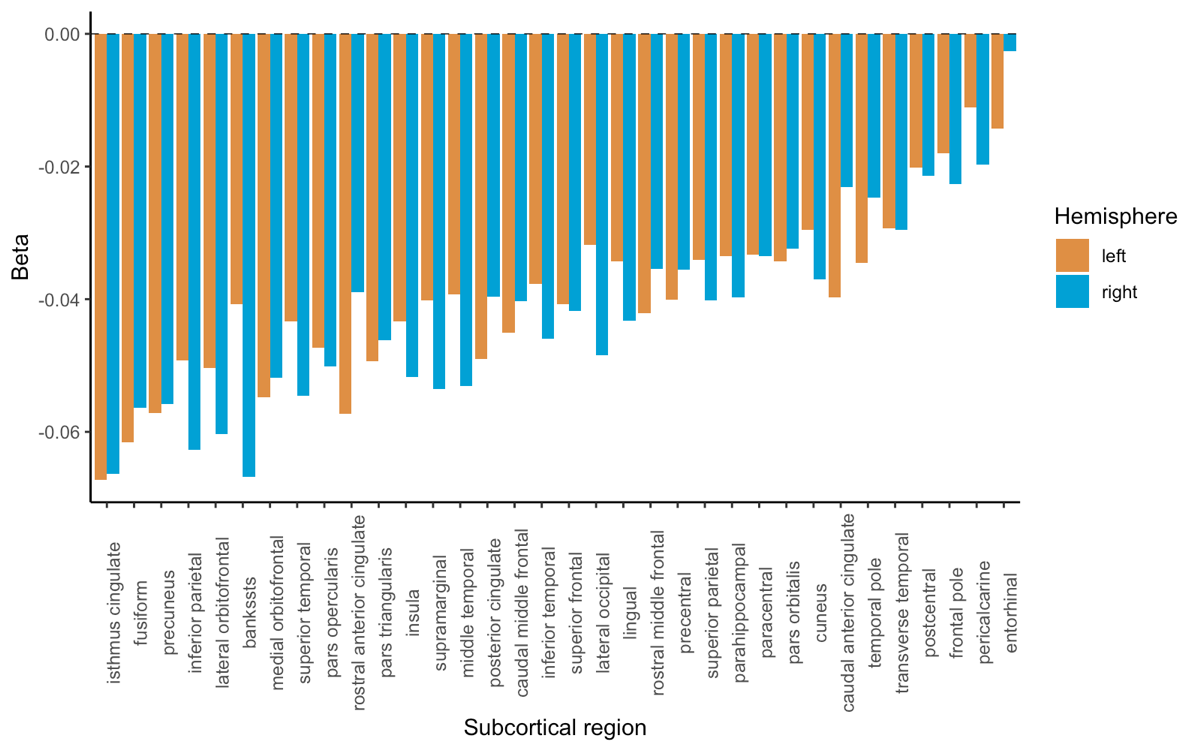


**eFigure 3. Longitudinal rates of change across cortical regions of interest.** All were statistically significant at *p*_FDR_ < 0.05 except for the right entorhinal region (*p*_FDR_ = 0.48) the left pericalcarine region (*p*_FDR_ = 0.058).

**eTable 2. Associations between risk factors and longitudinal atrophy.** Results derived from linear mixed-effects models. CI=confidence interval.

|  | **Beta (95% CI)** | ***p*-value** |
| --- | --- | --- |
| Intercept | 0.05 (0.30, 0.71) | <0.001 |
| Time | -0.07 (-0.08, -0.06) | <0.001 |
| Age at baseline | -0.5 (-0.63, -0.38) | <0.001 |
| Sex |  |  |
| Female | — |  |
| Male | -0.28 (-0.51, -0.06) | 0.013 |
| Charlson score | -0.02 (-0.12, 0.07) | 0.62 |
| Alcohol dependence at baseline | -0.13 (-0.36, 0.10) | 0.27 |
| Framingham score | -0.03 (-0.16, 0.10) | 0.63 |
| History of moderate or severe TBI | -0.27 (-0.47, -0.07) | 0.007 |
| Time × alcohol dependence | -0.05 (-0.07, -0.03) | <0.001 |
| Time × Framingham score | -0.02 (-0.03, -0.01) | <0.001 |


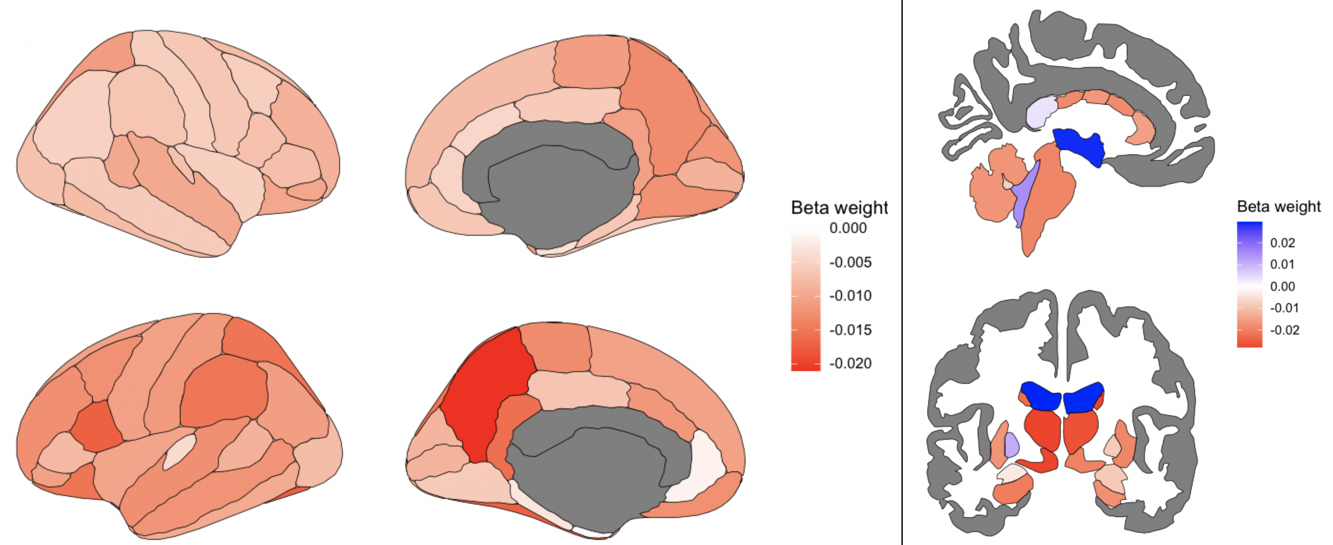


**eFigure 4. Rates of atrophy in those with alcohol dependence.** Predicted longitudinal trajectories of cortical thickness and subcortical structure volumes in individuals with alcohol dependence compared to those without alcohol dependence, adjusting for baseline age, sex, and estimated intracranial volume.


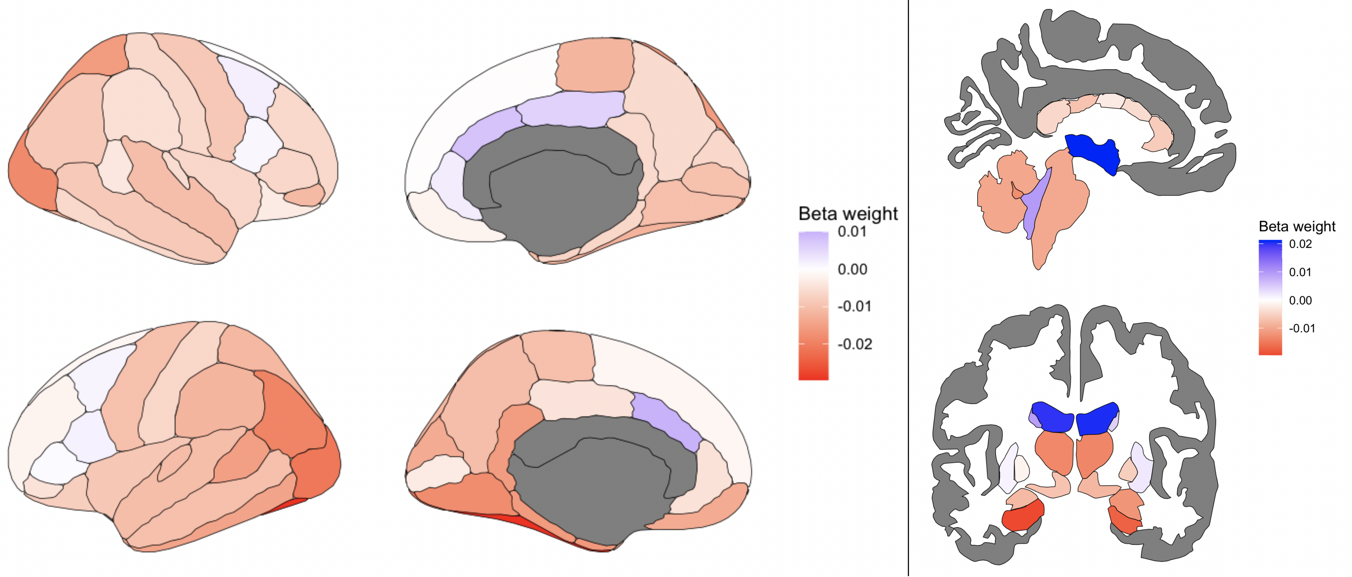


**eFigure 5. Rates of atrophy in those with higher Framingham risk scores.** Predicted longitudinal trajectories of cortical thickness and subcortical structure volumes in individuals with higher Framingham risk scores compared to those lower Framingham risk scores, adjusting for baseline age, sex, and estimated intracranial volume.

**eTable 3. Association between baseline atrophy and mortality during the study period.** HR=hazard ratio, CI=confidence interval, ref.=reference. Q1 (>75%) represents those with the least brain atrophy at baseline and Q4 (<25%) represents those with the most atrophy.

|  | **Unadjusted analysis** | | **Adjusted analysis** | |
| --- | --- | --- | --- | --- |
|  | **HR (95 % CI)** | ***p*-value** | **HR (95% CI)** | ***p*-value** |
| Age | — | — | 1.04 (1.01, 1.07) | 0.005 |
| Sex |  | | | |
| Female | — | — | ref. | ref. |
| Male | — | — | 0.96 (0.57, 1.63) | 0.9 |
| Charlson score | — | — | 1.08 (1.01, 1.16) | 0.021 |
| Atrophy quartile |  | | | |
| Q1 (>75%) | ref. | ref. | ref. | ref. |
| Q2 (50-75%) | 2.37 (1.06, 5.27) | 0.035 | 1.95 (0.86, 4.41) | 0.11 |
| Q3 (25-50%) | 4.01 (1.88, 8.53) | <0.001 | 2.74 (1.24, 6.08) | 0.013 |
| Q4 (<25%) | 4.91 (2.34, 10.3) | <0.001 | 2.51 (1.07, 5.86) | 0.034 |

**Supplementary references**

1. Reuter M, Schmansky NJ, Rosas HD, Fischl B. Within-subject template estimation for unbiased longitudinal image analysis. *NeuroImage*. 2012;61(4):1402-1418. doi:10.1016/j.neuroimage.2012.02.084

2. Desikan RS, Ségonne F, Fischl B, et al. An automated labeling system for subdividing the human cerebral cortex on MRI scans into gyral based regions of interest. *NeuroImage*. 2006;31(3):968-980. doi:10.1016/j.neuroimage.2006.01.021

3. Fischl B, Salat DH, Busa E, et al. Whole brain segmentation: automated labeling of neuroanatomical structures in the human brain. *Neuron*. 2002;33(3):341-355. doi:10.1016/s0896-6273(02)00569-x

4. Mowinckel AM, Vidal-Piñeiro D. Visualization of Brain Statistics With R Packages ggseg and ggseg3d. *Adv Methods Pract Psychol Sci*. 2020;3(4):466-483. doi:10.1177/2515245920928009

5. Endicott J. *Best Estimate Clinical Evaluation and Diagnosis Form (BECED)*. Department of Research Assessment and Training, New York State Psychiatric Institute; 1988.

6. Dams-OʼConnor K, Cantor JB, Brown M, Dijkers MP, Spielman LA, Gordon WA. Screening for traumatic brain injury: findings and public health implications. *J Head Trauma Rehabil*. 2014;29(6):479-489. doi:10.1097/HTR.0000000000000099
